# Adopting Basic Principles of the United Nations Academic Impact Initiative (UNAI): Can Cultural Differences Be Predicted from Value Orientations and Globalization?

**DOI:** 10.3389/fpsyg.2017.01977

**Published:** 2017-11-13

**Authors:** Andrea Nechtelberger, Walter Renner, Martin Nechtelberger, Soňa Chovanová Supeková, Maria Hadjimarkou, Chino Offurum, Panchalan Ramalingam, Birgit Senft, Kylie Redfern

**Affiliations:** ^1^Department of Psychology, Austrian Academy of Psychology, Vienna, Austria; ^2^Faculty of Psychology, Pan-European University, Bratislava, Slovakia; ^3^University of Nicosia, Nicosia, Cyprus; ^4^Faculty of Economics and Business, Pan-European University, Bratislava, Slovakia; ^5^Department of Social Sciences, University of Nicosia, Nicosia, Cyprus; ^6^Department for Religion, Culture, and Society, Whelan Research Academy, Owerri, Nigeria; ^7^Department of Applied Psychology, Pondicherry University, Pondicherry, India; ^8^UTS Business School, University of Technology Sydney, Sydney, Australia

**Keywords:** higher education, globalization, moral foundations theory, cultural virtues, United Nations Academic Impact Initiative

## Abstract

The United Nations Academic Impact (UNAI) Initiative has set forth 10 Basic Principles for higher education. In the present study, a 10 item self-report questionnaire measuring personal endorsement of these principles has been tested by self-report questionnaires with university and post-graduate students from Austria, China, Cyprus, India, Nigeria, and Slovakia (total *N* = 976, *N* = 627 female, mean age 24.7 years, *s* = 5.7). Starting from the assumptions of Moral Foundations Theory (MFT), we expected that personal attitudes toward the UNAI Basic Principles would be predicted by endorsement of various moral foundations as suggested by MFT and by the individual's degree of globalization. Whereas for the Austrian, Cypriot, and Nigerian sub- samples this assumption was largely confirmed, for the Chinese, Indian, and Slovak sub- samples only small amounts of the variance could be explained by regression models. All six sub-samples differed substantially with regard to their overall questionnaire responses: by five discriminant functions 83.6% of participants were classified correctly. We conclude that implementation of UNAI principles should adhere closely to the cultural requirements of the respective society and, where necessary should be accompanied by thorough informational campaigns about UN educational goals.

## Introduction

### The united nations academic impact initiative (“UNAI”)

UNAI is a worldwide network of higher education and research institutions who have agreed to promote 10 basic principles committed to human rights, equal chances, sustainability, global citizenship, and intercultural dialogue as expressed in Chapter 1 of the Charter of the United Nations (United Nations, 2015a). Such institutions are expected to cooperate closely both with UNAI and with each other in the course of scientific exchange of thoughts as well as collaborative research. These are the 10 basic principles of UNAI (UNAI, 2015a):

1. A commitment to the principles inherent in the United Nations Charter as values that education seeks to promote and help fulfill;
2. A commitment to human rights, among them freedom of inquiry, opinion, and speech;
3. A commitment to educational opportunity for all people regardless of gender, race, religion or ethnicity;
4. A commitment to the opportunity for every interested individual to acquire the skills and knowledge necessary for the pursuit of higher education;
5. A commitment to building capacity in higher education systems across the world;
6. A commitment to encouraging global citizenship through education;
7. A commitment to advancing peace and conflict resolution through education;
8. A commitment to addressing issues of poverty through education;
9. A commitment to promoting sustainability through education;
10. A commitment to promoting inter-cultural dialogue and understanding, and the “unlearning” of intolerance, through education (UNAI, 2015b).

The UNAI will celebrate its 5th anniversary in 2015. The UNAI was formally launched on the 18th of November 2010 at the UN headquarters in New York. The idea of the launch of UNAI was to encourage the mission of the United Nations of implementing the Millennium Development Goals, a set of eight targets. The goals respond to the world's challenges such as diseases, child mortality, primary education, poverty, gender equality and environmental sustainability (United Nations, 2015b).

UNESCO (United Nations Educational, Scientific and Cultural Organization) proclaims that education helps to build peace, to limit poverty, to foster lasting development and intercultural dialogue (UNESCO, 2015a). UNESCO developed a Global Citizenship Education Guide (UNESCO, 2015b) for the member states intended to be a resource for policy-makers and educators. The United Nations define certain values for education in their Academic Impact Initiative (UNAI). These values are the usefulness of the United Nations charter, a commitment to human rights, to equal opportunities, advancing peace through education and a few more.

Higher education institutes must play a more active role in the resolution of global challenges. The UNAI has its roots in fostering the idea of building stronger ties with institutions of higher learning (Ki-moon, 2008). Ki-moon introduces the Academic Impact concept as an additional tool to bring more attention to and focus on the United Nations work for peace, development and the protection of human rights. He demands the sharing of ideas, across borders and disciplines to find solutions for the world's challenges. The spirit behind the UNAI seeks to embrace and encourage a stronger culture of intellectual social responsibility (United Nations, 2010).

By now, more than 116 countries with 1,000 institutions have joined the initiative (UNAI, 2015b). The membership is open to all institutions of higher education as well as research bodies. Students are welcome to the ASPIRE (Action by Students to Promote Innovation and Reform through Education) initiative. ASPIRE connects student organizations and individuals with focus on the UNAI principles.

For each of the UNAI's 10 principles a corresponding global hub has been created. The purpose was to seek expression of interest for each theme, the sharing of best practices and activities and to develop a database of academic experts (United Nations, 2010). Ten universities were designated to promote the principles through initiatives, programs, information campaigns, publications or workshops: J. F. Oberlin University (Tokio), Sorbonne University (Paris), Lahore University of Management Sciences (Pakistan), George Mason University (United States), Handong Global University (South Korea), Ana G. Méndez University (Puerto Rico), University of Cetys (Mexico), University of Kwazulu-Natal (South Africa), Al Farabi Kazakh National University (Kasachstan), Escuela Politécnica Javeriana (Ecuador).

The academic impact initiative engages impressively the promotion of the United Nations work for the world's challenges in health, poverty, development of individuals, peace, environment and human rights. The hope is that more and more universities, institutions and their students will follow the spirit of the initiative, that education is a mission to sharing a culture of intellectual and social responsibility.

We have transformed the above mentioned 10 statements of the UNAI initiative into 10 items of a questionnaire, measuring an individual's endorsement of the 10 basic principles. The present study aimed at predicting the scores on this newly developed “UNAI questionnaire” (i.e., the attitude toward the UNAI Basic Principles) by moral and ethical values and by the personal degree of globalization.

#### Personal values as generalizations of attitudes toward UNAI

For the purpose of the present study, we define “culture” according to Richard A. Shweder, who conceptualized “cultural psychology” as “the study of the ways subject and object, self and other […] live together, require each other, and dynamically, dialectically, and jointly make each other up” (Shweder, 1999, p. 1).

As personal values (Renner et al., 2007) and the individual degree of globalization may be expected to differ between cultures, the study was carried out in Austria, China, Cyprus, India, Nigeria, and Slovakia; thus, communalities and differences within the predictive models of the six cultures should be assessed.

Human values and ethical positions related to them represent attitudes and personal goals on a higher level of abstraction: “Values are not the concrete goals of behavior, but rather are aspects of these goals. Values appear as the criteria against which goals are chosen, and as the implications which these goals have in the situation” (Kluckhohn, 1951, p. 429; cf., Schwartz, 1992). Following this relationship between attitudes and values, in the present study we tried to predict attitudes toward the UNAI Basic Principles from human values, expressed by the endorsement of “Moral Foundations” as conceptualized by Moral Foundations Theory (MFT).

#### A culture sensitive view of human values

Whereas traditional psychology was limited to a typically “Western” point of view, in the past decades cultural differences received growing attention. The concept of Individualism vs. Collectivism was introduced by Hofstede (1991) and Triandis (1995); along similar lines, Markus and Kitayama (1991) differentiated between the independent self of “Western,” individualist cultures and the interdependent self of collectivist, “non-Western cultures.” Hofstede (1991) added other dimensions which received comparatively less attention, namely: Power distance, uncertainty avoidance, masculinity vs. femininity, long-term vs. short-term orientation, and indulgence vs. restraint. Gelfand et al. (2011) added the cross-cultural dimension of looseness vs. tightness. Breaking new grounds methodologically, in an fMRI (Functional Magnetic Resonance Imaging) based study, Han et al. (2014) found substantial neurophysiological differences in moral decision making between participants from the USA and Korea.

Moral Foundation Theory was developed on the basis of an international review of the literature on the cultural variability of virtues (Haidt and Joseph, 2004). On this basis, Haidt and Kesebir (2010) developed five moral foundations, the endorsement of which varies between cultures:

1. Harm/care: Concerns for the suffering of others, including virtues of caring and compassion.
2. Fairness/reciprocity: Concerns about unfair treatment, cheating, and more abstract notions of justice and rights.
3. Ingroup/loyalty: Concerns related to obligations of group membership, such as loyalty, self- sacrifice, and vigilance against betrayal.
4. Authority/respect: Concerns related to social order and the obligations of hierarchicalrelationships, such as obedience, respect, and the fulfillment of role-based duties.
5. Purity/sanctity: Concerns about physical and spiritual contagion, including virtues of chastity, wholesomeness, and control of desires (Haidt and Kesebir, 2010, p. 822).

In terms of Shweder's (2008) cultural psychology, the first two foundations encompass an “Ethic of Autonomy”, Foundations 4 and 5 additionally focus on an “Ethic of Community”, and Foundation 5 is referred to by an “Ethic of Divinity.”

Graham et al. (2009) have found that among U.S. citizens, politically liberal respondents put high emphasis on Foundations 1 and 2, while neglecting Foundations 3, 4, and 5. U.S. Conservatives, as well as respondents from African and South Asian cultures, on the other hand, endorsed all five Moral Foundations to an equal extent.

Overall, following the culture-sensitive approach of MFT, we expected substantial differences between cultures with respect to their response patterns on the MFQ. Especially with student samples (who may be expected to be predominantly liberal with respect to their political convictions), marked differences between individualist and collectivist cultures may be expected. Members of individualist societies are concerned with avoiding harm to others and with behaving fairly especially toward the poor and underprivileged, whereas issues of identifying with one's group, obeying to authority, or of observing traditional or religious rules will be less important to them—as long as no objective harm is expected from the behavior in question. The opposite will be true with members of collectivist cultures, for whom violations of cultural rules are morally inacceptable, even if no objective harm results (e.g., using one's country flag as a bathroom rag; Haidt, 2001).

Therefore, although there are reports about the cultural invariance of the MFQ, e.g., between the US and New Zealand (Davies et al., 2014), for the “Western” part of the sample (i.e., Austria, Cyprus, and Slovakia), two-dimensional solutions were expected for the MFQ, the first dimension representing Foundations 1 and 2 and the second one representing Foundations 3, 4, and 5. We hypothesized that positive attitudes toward the UNAI Initiative would go along with higher scores on the first dimension and with lower scores on the second one. For the “Non-Western” part of the sample (i.e., China, India, and Nigeria) one-dimensional solutions were expected for the MFQ; we hypothesized that higher scores on this single dimension would go along with more positive attitudes toward the UNAI Initiative.

#### Globalization

In the developing world, especially young people are facing the challenge of globalization, i.e., the continuing unification of the globe with respect to clothing, language, nutrition, consumer decisions, media portrayals and consumption, to mention only a few aspects (cf., Renner et al., 2013 taking India as an example). Increasing globalization among youth may imply the risk of alienation from the older generations (Jensen et al., 2011) but can also promote self-esteem and open-mindedness (Eyou et al., 2000). Failure to accept the challenge of globalization may even be indicative of a fundamentalist retreat from the world (Almond et al., 1995).

According to Chiu and Hong (2006), there are marked individual differences with respect to the degree of personal identification with the issues of globalization. Whereas some individuals manage to combine the chances offered by globalization with their own cultural heritage, others fail to cope with this challenge. Overall, integrating global and ethnic values has been shown to be most beneficial with respect to psychological and socio-cultural outcomes (Berry, 2008).

These considerations led us to the expectation that a higher degree of globalization on the part of the individual will open his or her eyes to the concerns of the United Nations Academic Impact Initiative (UNAI). Thus, a higher degree of personal globalization was expected to predict a higher degree of consent with the Basic Principles of the UNAI Initiative. Again, we expected distinct response patterns for each culture.

## Methods

### Participants

In all six countries, respondents were recruited from universities or post-graduate educational institutes. The total sample comprised *N* = 976 participants, of which *N* = 627 were female. The mean age of the total sample was 24.7 years (*s* = 5.7 years, range 17–58 years). In Austria *N* = 246 students participated (*N* = 175 of them female, mean age 29.6 years, *s* = 7.8, range 19–50 years) and the Chinese sample comprised *N* = 155 respondents (*N* = 124 of them female, mean age = 20.6 years, *s* = 0.7, range 18–22 years). The Cypriot sample comprised *N* = 91 respondents (*N* = 71 female, mean age 24.4 years, *s* = 5.3, range 19–48 years); in India there were *N* = 155 participants (*N* = 109 female, mean age = 23.1 years, *s* = 2.1, range 20–33 years) and the Nigerian sample consisted of *N* = 227 participants (*N* = 88 female, mean age = 23.9 years, *s* = 3.9, range 17–38 years). In the Slovak sample there were *N* = 102 participants (*N* = 60 female, mean age = 23.3 years, *s* = 1.9, range 19–32 years).

### Questionnaires

#### UNAI questionnaire

This questionnaire comprises 10 items, each of them referring to one of the 10 Basic Principles of UNAI. The items can be endorsed on a five point Likert type scale, reaching from “Not at all important” (1) to “Completely important” (5). In addition, a “Don't know” response category was provided (coded as a “user defined missing” in the statistical analysis. The UNAI questionnaire is reprinted in the Appendix.

#### Globalization

The degree of personal globalization as assessed by a 12-item modified version (adapted for the respective culture) of the scale introduced by Redfern and Crawford (2010), addressing for example having friends from abroad, traveling abroad, knowledge of foreign language, preference for foreign food, western lifestyle, TV and movies, clothing etc. on an eight-point scale of agreement, ranging from 0 (= minimal) to 7 (= maximal).

#### Moral foundations

Endorsement of the Moral Foundations was assessed by the “Relevance” and “Judgment” section of the Moral Foundations Questionnaire (MFQ, Graham et al., 2009). The Relevance section comprises items like “Whether or not someone suffered emotionally” (Foundation 1) or “Whether or not someone violated standards of purity and decency” (Foundation 5). The instruction asks the participant to indicate the extent, to which each statement is personally relevant to the respondent by numbers ranging from 0 (“not all relevant”) to 5 (“extremely relevant”).

In the Judgment section of the MFQ, again comprising 10 items, respondents are asked to indicate by numbers ranging from 0 (“Strongly disagree”) to 5 (“Strongly agree”) the extent of their agreement. Item examples are: “When the government makes laws, the number one principle should be ensuring that everyone is treated fairly” (Foundation 2), “I am proud of my country's history” (Foundation 3), and “Respect for authority is something all children need to learn” (Foundation 4).

### Statistical methods

Responses to the questionnaire items were averaged across the respective scales, i.e., we computed arithmetic means, with the exception of response “6” (“Don't know”) to the UNAI questionnaire (see Appendix). According to the exploratory nature of the present study, the dimensionality of the MFQ and the Globalization scale was assessed by Exploratory Factor Analyses (EFA) in each sub-sample. The number of factors was determined on the basis of the variance explained by each of the factors, following the “scree” criterion after examining the eigenvalue-plots (Cattell, 1966).

In contrast, initially the UNAI Scale was assumed to possess “cultural invariance,” i.e., to measure equivalently across cultures. We examined this assumption by Confirmatory Factor Analysis (CFA, cf. section Measurement Invariance of the UNAI Scale Across Cultures).

## Results

The descriptive statistics obtained from the six sub-samples have been summarized in Table 1.

**Table 1:** Descriptive statistics of results.

**Country**	**UNAI**		**Globalization**		**MFQ relevance**		**MFQ judgment**	
	***M***	***SD***	***M***	***SD***	***M***	***SD***	***M***	***SD***
Austria	4.01	0.57	4.38	0.99	2.87	0.54	2.97	0.62
China	4.10	0.77	3.36	0.85	3.00	0.65	3.30	0.60
Cyprus	4.24	0.42	4.60	0.97	3.46	0.65	3.53	0.66
India	4.03	0.42	2.55	0.92	2.77	0.72	3.73	0.73
Nigeria	4.10	0.53	3.12	0.99	3.59	0.85	3.98	0.61
Slovakia	3.72	0.54	4.04	0.82	3.30	0.71	3.59	0.61

### Results for Austria

#### Scale characteristics

##### UN scale

Cronbach's alpha of this scale was 0.841, which is within acceptable limits. Reliability could not be further improved by eliminating items from the scale. Accordingly, Exploratory factor analysis (EFA, Principle Components Analysis with Varimax rotation) yielded a one- dimensional solution.

##### Globalization

Initially, Cronbach's alpha was 0.814, but could be improved to 0.817 by eliminating item 1 (recoded). EFA yielded three dimensions explaining a total of 60.7% of the variance.

Dimension 1 refers to personal experiences abroad or with people from other countries and thus was named “Experience,” Dimension 2, named “Interest” comprises items indicating personal interest in such experiences, and Dimension 3 (“English”) refers to the level of English spoken, written, or understood by the respondent.

##### Moral foundations questionnaire—relevance section

EFA^1^ yielded three dimensions, explaining 54.6% of the variance. Dimension 1, “Conservatism” refers to observing traditional values, related to authority and religion, Dimension 2, “Empathy” to caring for people in need, and Dimension 3, “Group welfare” comprises not betraying one's group, and not acting unfairly or in a disgusting way.

##### Moral foundations questionnaire—judgment section

In line with our expectations, by EFA two dimensions were extracted, which explained 42.7% of the variance. Dimension 1 (“Traditionalism”) comprises ethical judgments related to purity, authority, and respect, i.e., to higher principles which should be observed even if no other person would be harmed by an action infringing these ethical standards. Dimension 2 (“Liberalism”), on the other hand, addresses fairness, compassion, and avoiding harm to others.

#### Predicting scores on the UN-scale from globalization and moral standards

Apart from gender and age, the above-mentioned factor scores obtained from the globalization questionnaire (Experience, Interest, English), from the Relevance (Conservatism, Empathy, and Group welfare) and the Judgment section (Traditionalism and Liberalism) of the MFQ were entered into a linear regression model in order to predict the scores on the UN Scale.

Supporting our hypothesis, in the Austrian sample, this score was predicted by a high degree of Liberalism, foreign Experiences, and Empathy, by a higher age and by a lower degree of Traditionalism. The regression coefficients can be seen in Table 2.

**Table 2:** Linear regression model predicting the score on the UN-scale in the Austrian sample.

	**Non standardized coeffizients**		**Standardized coeffizients**		**Sig**.
	***B***	**Standard Error**	**Beta**	***t***	
(Constant)	3.783	0.178		21.203	0.000
Gender	−0.046	0.070	−0.036	−0.654	0.514
Age	0.010	0.004	0.141	2.479	0.014
Experience	0.089	0.033	0.155	2.718	0.007
Interest	0.055	0.032	0.096	1.741	0.083
English	−0.051	0.031	−0.088	−1.629	0.105
Conservatism	−0.058	0.036	−0.099	−1.602	0.111
Empathy	0.081	0.035	0.139	2.298	0.022
Group welfare	0.003	0.033	0.005	0.090	0.929
Traditionalism	−0.104	0.038	−0.178	−2.706	0.007
Liberalism	0.203	0.034	0.350	6.070	0.000

The remaining independent variables yielded no significant contribution to the prediction. Total *R*^2^ of the regression model was 0.34, indicating that 34% of the variance of the scores on the UN-Scale were explained.

### Results for China

#### Scale characteristics

##### UN scale

In the Chinese sample, for the internal consistency of the UN-Scale, Cronbach's alpha = 0.925 was computed. This result could not be improved any further by deleting items from the scale. EFA yielded a one-dimensional solution, which explained 60.4% of the variance.

##### Globalization

Initial Cronbach's alpha was 0.797. By deleting items 1 (reversed) and 7 from the scale, internal consistency could be further improved to 0.829. EFA again yielded a three-dimensional solution with respect to global ”Experience“ (Factor 1), ”Interest“ (Factor 2), and knowledge of ”English“ (Factor 3). The three factors explained 61.5% of the variance.

##### Moral foundations questionnaire—relevance section

Confirming our expectation for China's ”Non-Western“ culture, EFA yielded one dimension, which explained 31.9% of the variance. The respective scale ”Relevance“ had a Cronbach's alpha of 0.756, which could not be improved any further.

##### Moral foundations questionnaire—judgment section

Initially, according to the Scree Criterion, two dimensions were extracted. These were difficult to interpret on the basis of their content, however. Therefore, also in line with expectations, a one-dimensional solution was retained, which explained 28.8% of the variance. The respective scale had an alpha of 0.715, which could not be improved further.

#### Predicting scores on the UN-scale from globalization and moral standards

In the Chinese sample, higher scores on the UN-Scale were predicted by higher scores with respect to ”Interest“ in global affairs (*p* < 0.000), whereas the remaining independent variables did not contribute to the prediction. Overall, an *R*^2^ of only 0.099 was achieved. Details of the regression coefficients are reported in Table 3. Thus, in this respect, our hypothesis was not confirmed.

**Table 3:** Linear regression model predicting the score on the UN-scale in the Chinese sample.

	**Non standardized coeffizients**		**Standardized coeffizients**		**Sig**.
	***B***	**Standard Error**	**Beta**	***t***	
(Constant)	5.046	1.817		2.777	0.006
Gender	−0.201	0.165	−0.102	−1.214	0.227
Age	−0.047	0.086	−0.044	−0.547	0.585
Experience	−0.028	0.064	−0.037	−0.443	0.659
Interest	0.216	0.063	0.275	3.433	0.001
English	0.040	0.063	0.051	0.635	0.527
Relevance	0.093	0.109	0.076	0.846	0.399
Judgment	0.032	0.116	0.024	0.274	0.784

### Results for Cyprus

#### Scale characteristics

##### UN scale

With alpha 0.778, internal consistency of the scale was rather poor. It was improved to 0.783 by eliminating item 8. By EFA, two dimensions emerged, which explained 46.6% of the variance. The first component addresses issues of treating everybody equally and was named “Equality,” the second component mostly comprises items in the field of “Sustainability.”

##### Globalization

Initial Cronbach's alpha of this scale was 0.807. By eliminating Item 1 it was improved to 0.843. EFA yielded a four-factor solution, which explained 71.6% of the variance. Factor 1 was named “Experience” and Factor 2 addressed the command of “English.” Factor 3 referred to “Interest” in global matters and Factor 4 to the number of foreign ”Friends“ reported by the respondents.

##### Moral foundations questionnaire—relevance section

Two dimensions emerged in EFA, which explained 60.53% of the variance and which were in line with our hypothesis. Factor 1 again was called “Empathy” as it dealt with concerns of caring for people in need and acting fairly toward them, also including the importance of loyalty toward one's group. Factor 2, named “Conservatism,” on the other hand, comprised items addressing issues of authority, decency, respect, and purity.

##### Moral foundations questionnaire—judgment section

Again two factors were extracted which confirmed our expectations—Factor 1 “Traditionalism” and Factor 2 “Liberalism” with their content being comparable to the Austrian results. In this case, the two factors together explained 47.4% of the variance.

#### Predicting scores on the UN-scale from globalization and moral standards

##### Equality

The coefficients of the regression model for the prediction of the factor scores on “Equality” from the UN-Scale are summarized in Table 4.

**Table 4:** Linear regression model predicting the score on “Equality” from the UN-scale in the Cypriot sample.

	**Non standardized coeffizients**		**Standardized coeffizients**		**Sig**.
	***B***	**Standard Error**	**Beta**	***t***	
(Constant)	0.908	0.699		1.298	0.198
Gender	−0.367	0.245	−0.149	−1.500	0.138
Age	−0.010	0.018	−0.054	−0.575	0.567
Experience	0.059	0.094	0.060	0.635	0.527
Interest	−0.022	0.108	−0.022	−0.203	0.839
English	0.106	0.096	0.106	1.102	0.274
Traditionalism	−0.149	0.096	−0.150	−1.548	0.126
Empathy	0.244	0.108	0.231	2.260	0.027
Group welfare	0.086	0.125	0.080	0.682	0.497
Conservatism	−0.207	0.124	−0.194	−1.665	0.100
Liberalism	0.473	0.116	0.465	4.059	0.000

In the Cypriot sample, in line with expectations, high factor scores and on “Liberalism” and “Empathy” predicted higher values on the UN-Scale, whereas the other independent variables did not contribute significantly to the prediction. In this case, the total *R*^2^ amounted to 0.402.

##### Sustainability

High factor scores on Sustainability were only predicted by a higher level of experience with other cultures. The details of the regression model with a total *R*^2^ = 0.189 are given in Table 5.

**Table 5:** Linear regression model predicting the score on “Sustainability” from the UN-scale in the Cypriot sample.

	**Non standardized coeffizients**		**Standardized coeffizients**		**Sig**.
	***B***	**Standard Error**	**Beta**	***t***	
(Constant)	−0.548	0.807		−0.679	0.499
Gender	−0.301	0.283	−0.123	−1.065	0.290
Age	0.046	0.020	0.249	2.270	0.026
Experience	0.256	0.108	0.259	2.369	0.020
Interest	−0.240	0.124	−0.245	−1.931	0.057
English	0.036	0.110	0.036	0.322	0.748
Conservatism	0.170	0.111	0.173	1.529	0.130
Empathy	−0.076	0.125	−0.072	−0.607	0.546
Group welfare	−0.135	0.145	−0.128	−0.935	0.353
Traditionalism	−0.011	0.143	−0.011	−0.077	0.938
Liberalism	0.017	0.134	0.017	0.127	0.900

Thus, our hypotheses regarding the predictive power of MFQ scores and globalization were only partly confirmed in the Cypriot sample.

### Results for India

#### Scale characteristics

##### UN scale

The internal consistency of the total scale was alpha = 0.609 and could not be further improved by eliminating items. EFA yielded two dimensions, which together explained 38.5% of the variance. Factor 1 deals with sustainability and resources for the poor (“Sustainability”), whereas Factor 2 addresses issues of justice and equal opportunities for everybody (“Equality”).

##### Globalization

For the total scale, initial Cronbach's alpha was 0.740. Eliminating items in order to improve internal consistency would have led to a loss of approximately half of the total number of items. EFA yielded three factors, which explained 62.7% of the variance. Factor 1 again was termed “Experience,” Factor 2 “Interest,” and Factor 3 “English” as in the previous sub- samples.

##### Moral foundations questionnaire—relevance section

In line with our expectation, EFA yielded only one clearly interpretable dimension, which explained 28.6% of the variance and was termed for “Relevance.” For the respective scale an alpha = 0.710 was computed, which could not be improved further by eliminating items from the scale.

##### Moral foundations questionnaire—judgment section

Only one clearly interpretable dimension was retained, although by the Scree Criterion two dimensions would have been suggested. Thus, again our expectation of a one-dimensional solution for the Indian sample was confirmed. This dimension explained 33.3% of the variance. When a total scale for “Judgment” was formed, Cronbach's alpha was 0.738. By eliminating items 5 and 7, internal consistency could be improved to alpha = 0.786.

#### Predicting scores on the UN-scale from globalization and moral standards

##### Sustainability

As can be seen from Table 6, and in line with expectations, higher scores on “Interest” in global affairs and on the “Judgment” section of the MFQ were highly significant predictors of the “Sustainability” Score of the UN-Scale, whereas the other dimensions of globalization did not contribute to the prediction. Total *R*^2^ was 0.199, indicating that about 20% of the variance were explained by the independent variables.

**Table 6:** Linear regression model predicting the score on “Sustainability” from the UN-scale in the Indian sample.

	**Non standardized coeffizients**		**Standardized coeffizients**		**Sig**.
	***B***	**Standard Error**	**Beta**	***t***	
(Constant)	−1.082	1.065		−1.015	0.312
Gender	0.058	0.172	0.026	0.335	0.738
Age	−0.020	0.036	−0.043	−0.567	0.571
Experience	−0.027	0.082	−0.027	−0.333	0.739
Interest	0.257	0.076	0.257	3.376	0.001
English	0.073	0.076	0.073	0.959	0.339
Relevance	−0.013	0.110	−0.010	−0.122	0.903
Judgment	0.404	0.114	0.296	3.552	0.001

##### Equality

In this case, none of the independent variables yielded significant predictions of the dependent variables and thus, our hypothesis was disconfirmed.

### Results for Nigeria

#### Scale characteristics

##### UN scale

In the Nigerian sample, Cronbach's alpha for the UN-Scale was 0.785. EFA yielded a one- dimensional solution, explaining 34.8% of the variance.

##### Globalization

For this scale, initial alpha was 0.643, which could be improved to only 0.702 by removing Item Nr. 1 (recoded). EFA yielded a three-factor solution which explained 52.4% of the variance. Dimension 1 comprised foreign “Experience,” dimension 2 the respondents' level of “English,” and Dimension 3 their “Interest” in global affairs.

##### Moral foundations questionnaire—relevance section

In line with the expectations, EFA yielded a one-dimensional solution, explaining 42.9% of the variance. For this one-dimensional scale, Cronbach's alpha was 0.850 and could not be improved further by deleting items from the scale.

##### Moral foundations questionnaire—judgment section

Again, by EFA, confirming our hypothesis, only one dimension was found, which explained 27.3% of the variance. According to the Scree plot, a second dimension could be extracted, but did not make sense with respect to its content. Cronbach's alpha of this scale was 0.682 and could not be improved by eliminating items.

#### Predicting scores on the UN-scale from globalization and moral standards

In the Nigerian sample, in line with our hypothesis, higher scores on the UN Scale were predicted highly significantly by higher scores on the Relevance and Judgment dimensions of the MFQ. Total *R*^2^ was 0.248. The regression coefficients can be seen from Table 7. The sub- scales of Globalization, however, did not contribute significantly to the prediction.

**Table 7:** Linear regression model predicting the score on the UN-scale in the Nigerian sample.

	**Non standardized coeffizients**		**Standardized coeffizients**		**Sig**.
	***B***	**Standard Error**	**Beta**	***t***	
(Constant)	2.404	0.331		7.261	0.000
Gender	0.015	0.073	0.014	0.201	0.841
Age	0.002	0.009	0.014	0.205	0.838
Experience	0.056	0.031	0.105	1.771	0.078
English	0.045	0.032	0.084	1.395	0.164
Interest	0.025	0.032	0.048	0.802	0.423
Relevance	0.160	0.040	0.258	3.994	0.000
Judgment	0.265	0.054	0.304	4.902	0.000

### Results for Slovakia

#### Scale characteristics

##### UN scale

In this sub-sample, Cronbach's alpha initially was 0.691 and could not be improved any further. EFA yielded a one-dimensional solution which explained 26.2% of the variance.

##### Globalization

Initial internal consistency was 0.691 and could be improved to 0.720 by eliminating item 1 (reversed). By EFA three meaningful dimensions were extracted which explained 53.2% of the variance. Factor 1 comprised items referring to international “Experience,” whereas Factor 2 addressed the level of command of “English,” and Factor 3 referred to the persons “Interest” in global affairs.

##### Moral foundations questionnaire—relevance section

In this case, as hypothesized, two dimensions explaining 48.7% of the variance were found to be the optimal solution. Factor 1, named “Conservatism” was concerned with decency, authority, and tradition, whereas Factor 2, named “Liberalism” addressed the importance of avoiding harm to others and fairness.

##### Moral foundations questionnaire—judgment section

Contrary to expectations, however, a one-dimensional solution which explained 25.7% of the variance was chosen as suggested by the scree plot. Accordingly, two- or three-dimensional solutions did not yield interpretable results. For the resulting scale, Cronbach's alpha was 0.628 and could not be improved any further.

#### Predicting scores on the UN-scale from globalization and moral standards

Relatively high scores on the Judgment dimension from the MFQ predicted higher scores on the UN-Scale, whereas none of the other independent variables yielded significant contributions to the prediction. Thus, our hypotheses regarding the predictive power of MFQ scores and globalization were only partly confirmed. Total *R*^2^ amounted only to 0.133. Table 8 summarizes the regression coefficients.

**Table 8:** Linear regression model predicting the score on the UN-scale in the Slovakian sample.

	**Non standardized coeffizients**		**Standardized coeffizients**		**Sig**.
	***B***	**Standard Error**	**Beta**	***t***	
(Constant)	4.508	0.734		6.144	0.000
Gender	−0.052	0.116	−0.048	−0.450	0.654
Age	−0.030	0.029	−0.105	−1.034	0.304
Experience	−0.048	0.056	−0.090	−0.866	0.389
English	0.065	0.055	0.122	1.194	0.236
Interest	0.067	0.053	0.125	1.263	0.210
Conservatism	0.073	0.053	0.137	1.375	0.173
Liberalism	0.052	0.056	0.097	0.930	0.355
Judgment	0.122	0.056	0.227	2.171	0.033

### Measurement invariance of the UNAI scale across cultures^2^

We examined cross-cultural measurement invariance by Confirmatory Factor Analysis (CFA), assuming that all the 10 items of the scale would measure an identical construct. This assumption was tested by the Chi-Square test of significance as well as by the fit indices CMIN/df (Wheaton et al., 1977), TLI (Tucker-Lewis Index, Tucker and Lewis, 1973), CFI (Comparative Fit Index, Hu and Bentler, 1999), and RMSEA (Root mean square error of approximation, Hooper et al., 2008). CFA was performed by the AMOS software (Arbuckle, 2014) and the results interpreted following the guidelines by Weiber and Mühlhaus (2010).

As can be seen from Table 9, model fit for the total sample is not satisfactory *(CMIN* = 367.6; *df* = 35; *p* < 0.001; *CMIN/df* = 10.50; *TLI* = 0.767; *CFI* = 0.852; *RMSEA* = 0.099; factorial reliability = 0.82; *DEV* = 0.32). Taking the different cultures into account, even the unconstrained model has a worse model fit, with the only exception of RMSEA *(CMIN* = 631.09; *df* = 210; *p* < 0.001; *CMIN/df* = 3.005; *TLI* = 0.724; *CFI* = 0.824; *RMSEA* = 0.045). Thus, configural invariance has not been achieved and, applying the stricter criteria of metric and scalar invariance, model fit would deteriorate even further.

**Table 9:** Measurement invariance of the UNAI-scale across cultures.

**Model**	**CMIN**	***df***	***p***	**CMIN/df**	**TLI**	**CFI**	**RMSEA**
Total	367.6	35	<0.001	10.50	0.767	0.852	0.099
Unconstrained	631.09	210	<0.001	3.005	0.724	0.824	0.045
Measurement weights	769.89	260	<0.001	2.961	0.730	0.787	0.049
Measurement intercepts	1,235.96	310	<0.001	3.987	0.588	0.613	0.059
Measurement residuals	1,553.17	360	<0.001	4.314	0.543	0.502	0.055

### Comparison of the six participating cultures

Due to the different dimensionality of the scales in each culture, this comparison had to be done on the item level. In order to find cultural differences and characteristics, discriminant analysis has been employed. By the five discriminant functions, 83.6% of the individuals could be classified correctly. Details of correct and false classifications can be seen from Table 10.

**Table 10:** Results of classification by discriminant analysis.

**Predicted group membership**								
	**Country**	**Austria**	**India**	**Nigeria**	**China**	**Cyprus**	**Slovakia**	**Total**
N	Austria	104	0	1	3	2	10	120
	India	0	97	12	7	2	1	119
	Nigeria	1	17	187	3	10	9	227
	China	2	5	3	123	7	3	143
	Cyprus	0	1	2	2	46	4	55
	Slovakia	5	0	2	4	2	56	69
%	Austria	86.7	0.0	0.8	2.5	1.7	8.3	100.0
	India	0.0	81.5	10.1	5.9	1.7	0.8	100.0
	Nigeria	0.4	7.5	82.4	1.3	4.4	4.0	100.0
	China	1.4	3.5	2.1	86.0	4.9	2.1	100.0
	Cyprus	0.0	1.8	3.6	3.6	83.6	7.3	100.0
	Slovakia	7.2	0.0	2.9	5.8	2.9	81.2	100.0

*243 cases excluded because of missing data*.

**Figure 1:**
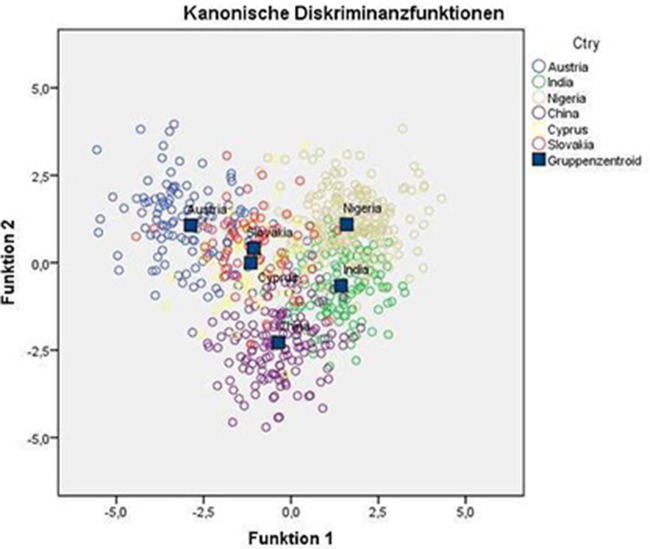
Scatterplot of discriminant functions 1 and 2: group differences.

Discriminant Function 1 explained 48.2%, Function 2 explained 29.7%, Function 3 10.0%, Function 4 explained 6.9%, and Function 5 explained 5.1% of the variance. The respective eigenvalues were 2.7, 1.7, 0.6, 0.4, and 0.3. With respect to the low amount of variance explained by Discriminant Functions 3–5, a scatterplot taking only Functions 1 and 2 into account gives a fair impression of the group differences but still should be interpreted with caution, especially at the single-item level. This diagram is given in Figure 1.

## Discussion

Our basic assumption was that the six cultures would differ with respect to their overall response pattern. This expectation clearly was confirmed by the result of discriminant analysis which allowed us to separate the sub-samples clearly from each other and to allocate 83.6% of respondents correctly according to the discriminant functions. From Figure 1 it can be seen that the sub-samples can be differentiated clearly even on the basis of only the first two discriminant functions. In an attempt to interpret the diagram in more detail, it can be seen that the countries are rank ordered by their *per capita* gross domestic product (GDP, 2010-2014) on Function 1: (1) Austria (with a GDP of $ 46,164), (2) Cyprus ($ 30,873), (3) Slovakia ($ 27,584), (4) China ($ 13,216), (5) India ($ 5,833), and (6) Nigeria ($ 967) (The World Bank, 2015). Per capita GDP has been shown to correlate substantially with individualism as opposed to collectivism (Gorodnichenko and Roland, 2010).

Conversely, when examining the graph along its upper right to lower left diagonal, it can be seen that the countries are rank ordered fairly correctly according to their ”Long Term Orientation“ (LTO) which has been defined as follows: ”Societies who score low on this dimension, for example, prefer to maintain time-honored traditions and norms while viewing societal change with suspicion. Those with a culture which scores high, on the other hand, take a more pragmatic approach: they encourage thrift and efforts in modern education as a way to prepare for the future“ (Hofstede, 2015a). Nigeria, located in the upper right part of the graph, scores lowest (LTO = 13), whereas China, positioned in the lower left, scores highest (LTO = 87), with the remaining samples lying in between (Austria: LTO = 60; Cyprus: LTO = 45^3^; India: LTO = 51; Slovakia: LTO = 77; Hofstede, 2015b). Though this interpretation may be somewhat speculative, it may at least be concluded that the sub-samples can be differentiated from each other in a meaningful way.

From the results, it became also clear that the dimensionality of the UNAI-Scale as well as that of the dependent variables varies between cultures. In the Austrian, Chinese, Nigerian, and Slovak sub-samples, the UNAI-Scale was one-dimensional, whereas in the Cypriot and Indian samples two dimensions emerged, one referring to equality, and one to sustainability. Thus, it did not come as a surprise that measurement invariance of the UNAI-Scale could not be established across cultures by Confirmatory Factor Analysis.

In line with MFT, in the Austrian and Cypriot sample, both sections of the Moral Foundations Questionnaire yielded at least two dimensions, one referring to conservative or traditional values, the other one to empathy and to values of doing no harm to others and caring for the poor. In line with MFT, in the Chinese, Indian, and Nigerian samples, participants did not distinguish between “conservative” and “liberal” values or moral foundations and one-dimensional solutions resulted for the MFQ. Surprisingly, in the Slovak sub-sample, the expected two-dimensional solution for the MFQ was only found with regard to the Relevance- but not for the Judgment-Section of the MFQ.

We had also assumed that the scores on the UNAI scale could be predicted by the personal degree of globalization. For the “Western” sub-samples in Austria, Cyprus, and Slovakia, “Liberalism” as opposed to “Conservatism” or “Traditionalism” was expected to predict higher scores on the UNAI scale. This hypothesis was confirmed for Austria and Cyprus, but not for Slovakia.

In the “Non-Western” sub-samples the one-dimensional MFQ scores were expected to predict endorsement of the UNAI scale. Whereas this expectation was confirmed for the Nigerian and—though only in part—for the Indian sub-samples, no successful predictions could be made for the Chinese sub-sample.

In all six sub-samples, for the Globalization Scale, three dimensions were found, one referring to global experience, one to global interests, and one to the level of English spoken and written. Only in the Cypriot sample, there was a fourth dimension, referring to having friends from abroad. In the Austrian and the Cypriot sample, global experience predicted UNAI scores, and the same was the case for global interest in the Chinese and Indian samples. This can be well understood by the fact that respondents from developing countries have fewer opportunities to make “real world” global experiences and thus their global interests may be more predictive than their experiences.

Overall, when taking into consideration the percentage of variance explained, acceptable predictions were made for Austria (34%), Cyprus (40 and 19% for the two dimensions of the UNAI scale respectively), and Nigeria (25%). Predictions for China (10%), India (20% for the “Sustainability” sub-scale of the UNAI questionnaire, but no prediction for the “Equality” sub-scale), and Slovakia (13%), however, were surprisingly poor. Clearly, these differences cannot be accounted for by the six cultures' positions along a “West-East” or an “individualist-collectivist” continuum, but may reflect different degrees of previous information about UN educational goals on the part of the respondents. This conclusion is supported by the observation that the amounts of variance explained are correlated positively and substantially with the respective cultures' globalization scores (*r* = 0.613).

In summary, when interpreting results, two aspects must be differentiated:

1. *Possible predictors:* as in the “Western” or “individualist” part of the world, moral attitudes (personal values) align along two dimensions, namely “Liberalism” and “Conservatism/Tradition,” these two dimensions are possible predictors of UNAI scores; in “Non-Western” or “Collectivist” cultures, however, moral attitudes (personal values) may be expected to be one-dimensional and thus only one dimension, i.e., their overall arithmetic mean should be expected.
2. *Predictability of UNAI scores from personal values (moral attitudes):* in less globalized cultures which—at present—may be less familiar with UN educational goals, only small amounts of the variance of UNAI scores may be predicted from personal values (moral attitudes).

Limitations of the present study pertain to the restricted number of cultures included. Taking only three “Western” and three “Non-Western” cultures into consideration, does not suffice to warrant safe generalizations. Another limitation concerns selection of the samples. When addressing educational values, not only students', but also their professors' and their parents' attitudes and values should be taken into consideration and ideally, should be followed up over several years in a longitudinal design. Moreover, even within the same culture, across different academic populations, liberal vs. traditional positions may vary with respect to their dimensionality and extent—especially when considering various study majors.

Additional limitations result from the theoretical framework of the study which was derived from the distinction of individualist vs. collectivist cultures and from Richard A. Shweder's Cultural Psychology. Future studies might gain new insights by taking political and economic differences between cultures into account and develop more differentiated hypotheses on the basis of such an interdisciplinary approach.

Apart from encouraging further research which might take these possibilities into account, the present exploratory study yields suggestions which might be of high importance for the practical implementation of the UNAI policy: we have found marked cultural differences with respect to the differential reception and understanding of UNAI by persons from different cultures. The results also suggest that in the “Western” part of the world, “liberals” may be more likely to attend to UNAI standards than “conservatives.”

When implementing UNAI policy, cultural specificities and peculiarities should be taken into consideration. The results suggest that UNAI standards should be communicated by local experts who are knowledgeable about the value systems of the respective culture. They should be ready to adapt these standards to local requirements and expectations in a culturally sensible way.

## Ethics statement

The Ethics committee of the Austrian Academy of Psychology approved the study. Each participant was provided with all the details and explanations, in simple language, regarding the participation in the study. The participant was informed on the information and/or material and who will have access to this information and material. The duration, for which the investigators will have access to the information and/or material that the respondent will provide, was be specified. The aim of the study was explained and what is hoped to be achieved as a result of the participation. The adherence of confidentiality and anonymity of the participant was ensured. The survey was conducted so that no conclusions on individual persons may arise. The participation was totally voluntary. At any point of time, for the participant it was possible to withdraw without any penalty. A consent form was signed by the participants.

## Author contributions

AN: Project lead. WR: Methods and statistics lead. BS: Additional statistics. All other authors: data collection, comments, and improvements.

### Conflict of interest statement

The authors declare that the research was conducted in the absence of any commercial or financial relationships that could be construed as a potential conflict of interest.

## Footnotes

^1^Detailed results from exploratory factor analyses are available upon request.

^2^Detailed results from confirmatory factor analyses are available upon request.

^3^Data from Greece, as Cyprus is missing from Hofstede's data base.
