# Supplementary material for: Adopting Basic Principles of the United Nations Academic Impact Initiative (UNAI): Can Cultural Differences Be Predicted from Value Orientations and Globalization?
Source: Front Psychol. 2017 Nov 13;8:1977. doi: 10.3389/fpsyg.2017.01977 (PMC5693894; doi:10.3389/fpsyg.2017.01977)
Supplement: Supplementary file 1 [file Appendix.pdf]

Survey Based on the United Nations Academic Impact Initiative

Dear student!

The United Nations Academic Impact is informed by a commitment to support and advance ten basic principles.

The University of Nicosia is conducting a survey on the importance of various educational principles for your student life.

(1) First, please fill out some demographic information:

gender: ☐ male ☐ female **age:** ________ years old

(2) Please indicate the degree of personal importance of the following statements for you at the present time. Please be sure to use the full scale of possible answers *(some statements should be clearly less important than others!!!)*:

	Not at all important	Slightly important	Moderately important	Very important	Completely important	Don't know
1. Education shall promote and fulfill the values of the United Nations charter.	1	2	3	4	5	6
2. Commitment to human rights (e.g. freedom of inquiry, opinion and speech) is important.	1	2	3	4	5	6
3. Equal educational opportunity, regardless of gender, race, religion or ethnicity is important.	1	2	3	4	5	6
4. Every interested individual should have the opportunity to acquire skills and knowledge necessary for the pursuit of higher education.	1	2	3	4	5	6
5. Capacity building in higher education systems across the world is important.	1	2	3	4	5	6

	Not at all important	Slightly important	Moderately important	Very important	Completely important	Don't know
6. Education encourages global citizenship.	1	2	3	4	5	6
7. Peace and conflict resolution advance through education.	1	2	3	4	5	6
8. Issues of poverty can be addressed through education.	1	2	3	4	5	6
9. Sustainability can be promoted through higher education.	1	2	3	4	5	6
10. Intercultural dialogue, understanding and tolerance can be promoted through education.	1	2	3	4	5	6

(3) We wish to assess the degree of "globalization" in our respondents. We therefore ask you to give the following ratings on the 7-point scale provided (please compare yourself with average same-gender Cypriots of your age):

	Min-imal							Max-imal
1. Your self-evaluation of traditional Cypriot values	0	1	2	3	4	5	6	7
2. Your level of spoken English	0	1	2	3	4	5	6	7
3. Your level of written English	0	1	2	3	4	5	6	7
4. Your interest in non-Cypriot movies or TV programs	0	1	2	3	4	5	6	7
5. Your interest in non-Cypriot fashion or clothes	0	1	2	3	4	5	6	7
6. Your interest in eating or cooking non-Cypriot food	0	1	2	3	4	5	6	7
7. Your interest in travelling to non-Cypriot countries	0	1	2	3	4	5	6	7
8. Your amount of time already spent outside Cyprus	0	1	2	3	4	5	6	7
9. Your amount of time already spent in English speaking countries	0	1	2	3	4	5	6	7
10. Your amount of time already spent in other countries	0	1	2	3	4	5	6	7
11. Your degree of friendship to non-Cypriot colleagues or peers	0	1	2	3	4	5	6	7
12. The estimated number of non-Cypriot friends	None	1-4	5	6-9	10	11-20	20	> 20

(4) Moral Foundations Questionnaire

Part A. When you decide whether something is right or wrong, to what extent are the following considerations relevant to your thinking? Please rate each statement using this scale:

[0] = not at all relevant (This consideration has nothing to do with my judgments of right and wrong)
[1] = not very relevant
[2] = slightly relevant
[3] = somewhat relevant
[4] = very relevant
[5] = extremely relevant (This is one of the most important factors when I judge right and wrong)

______Whether or not someone suffered emotionally
______Whether or not some people were treated differently than others
______Whether or not someone’s action showed love for his or her country
______Whether or not someone showed a lack of respect for authority
______Whether or not someone violated standards of purity and decency
______Whether or not someone was good at math
______Whether or not someone cared for someone weak or vulnerable
______Whether or not someone acted unfairly
______Whether or not someone did something to betray his or her group
______Whether or not someone conformed to the traditions of society
______Whether or not someone did something disgusting

Part B. Please read the following sentences and indicate your agreement or disagreement:

[0]	[1]	[2]	[3]	[4]	[5]
Strongly disagree	Moderately disagree	Slightly disagree	Slightly agree	Moderately agree	Strongly agree

______Compassion for those who are suffering is the most crucial virtue.
______When the government makes laws, the number one principle should be ensuring that everyone is treated fairly.
______I am proud of my country’s history.
______Respect for authority is something all children need to learn.
______People should not do things that are disgusting, even if no one is harmed.
______It is better to do good than to do bad.
______One of the worst things a person could do is hurt a defenseless animal.
______Justice is the most important requirement for a society.
______People should be loyal to their family members, even when they have done something wrong.
______Men and women each have different roles to play in society.
______I would call some acts wrong on the grounds that they are unnatural.

The Moral Foundations Questionnaire (short version, July 2008) by Jesse Graham, Jonathan Haidt, and Brian Nosek.
